# From biofilm control to biomimetic remineralization: Hydrogels in prevention and treatment of dental caries

**DOI:** 10.3389/fcimb.2025.1663563

**Published:** 2025-09-17

**Authors:** Yuqing Chen, Shikang Lin, Xiaojing Huang, Wen Zhou

**Affiliations:** Fujian Key Laboratory of Oral Diseases and Fujian Provincial Engineering Research Center of Oral Biomaterial and Stomatological Key Lab of Fujian College and University, School and Hospital of Stomatology, Fujian Medical University, Fuzhou, China

**Keywords:** dental caries, hydrogel, antibacterial, remineralization, prevention and treatment

## Abstract

Dental caries, a prevalent chronic bacterial disease globally, poses a significant threat to public health due to its complex pathogenesis involving demineralization and microbial dysbiosis. Hydrogels, with their unique three-dimensional network structures and diverse properties, have shown great potential in prevention and treatment of dental caries. This article systematically reviews recent advances in anti-caries hydrogel development. It first introduces the basis of anti-caries hydrogels, covering the applications of natural and semi-synthetic polymers as hydrogel matrices. Then, it elaborates on the mechanisms and research status of different types of anti-caries hydrogels, including probiotic formulations, antibacterial hydrogels, remineralization-inducing hydrogels, and saliva-related caries-reducing hydrogels. Finally, it summarizes the current research achievements and limitations and looks ahead to future research directions.

## 1 Introduction

Dental caries is a chronic bacterial disease affecting the hard tissue of the teeth and is recognized as a significant, escalating global public health challenge. According to the World Health Organization (WHO) in 2023, dental caries is the most prevalent non-communicable chronic disease worldwide. Over one-third of the global population living with untreated caries (Benzian et al., 2022). The development of dental caries results from a complex interplay over time among acid-producing bacteria and fermentable carbohydrates and host factors, including the condition of teeth and saliva (Marsh, 1994; Featherstone, 2004; Mandel, 1989). The pathological changes involve the organic matter decomposition and inorganic component demineralization in dental hard tissues (Wefel et al., 1985). Targeting the key factors lead to dental caries formation is effective in preventing and treating this chronic disease.

Antibacterial materials, competitive oral probiotics, and remineralization promoters are increasingly studied for synergistic caries management (Selwitz et al., 2007). Nowadays, quite a lot of improved anti-caries hydrogels are studied and developed, by taking use of anti-caries agents (Jenkins, 1985; Krasse, 1988; Rafiee et al., 2024; Zhen et al., 2022). Complex physicochemical oral environments and bacterial biofilms are considered critical factors compromising the clinical performance of anti-caries materials (Paula and Koo, 2017; Chen et al., 2016). Hydrogel application can enhance key properties of anti-caries agents and improve their effectiveness, such as prolonging duration of action and retention at target sites (Senel et al., 2000). This attribute to hydrogels’ three-dimensional networks that possess properties including electroconductivity, swellability, environmental sensitivity, and viscosity (Jang et al., 2021; Lei et al., 2023; Zhang et al., 2020; Chen et al., 2023; Liang et al., 2021; Vinikoor et al., 2023). The strong hydrophilicity enables hydrogel remain stable for a long time after swelling in water (Thang et al., 2023). This stability enhances both the duration of action and site retention compared to traditional materials (Li et al., 2024). Moreover, hydrogels’ cross-linked polymeric structure provides three-dimensional stability that enables effective loading of active ingredients while serving as a remineralization scaffold (Thang et al., 2023; Ding et al., 2022). These features make hydrogel promising material for caries management. Integrating traditional materials with hydrogels significantly enhances caries preventive and therapeutic efficacy (Rafiee et al., 2024; Zhen et al., 2022; Fathy et al., 2024; Cao et al., 2014; Estes Bright et al., 2022; Matsuda et al., 2022).

In recent years, hydrogels were increasingly studied for caries prevention and treatment (Muşat et al., 2021; Cai and Moradian-Oldak, 2023; Zhang et al., 2021c). Generally, hydrogels are composed of main body, serving as the carrier, and functional payloads. Consequently, hydrogels can be classified into natural and semi-synthetic types based on their structural composition (Loessner et al., 2016; Ho et al., 2022; Corkhill et al., 1989). The loaded ingredients endow the hydrogels with specific functions: antibacterial action, remineralization, probiotic delivery, and reducing saliva-related caries. Therefore, the present paper reviews hydrogel application in caries control, analyze their preventive/therapeutic roles, and outlines future research directions. Additionally, current challenges and potential solutions for complex hydrogels are discussed.

## 2 The base of anti-caries hydrogels

**Figure 1:**
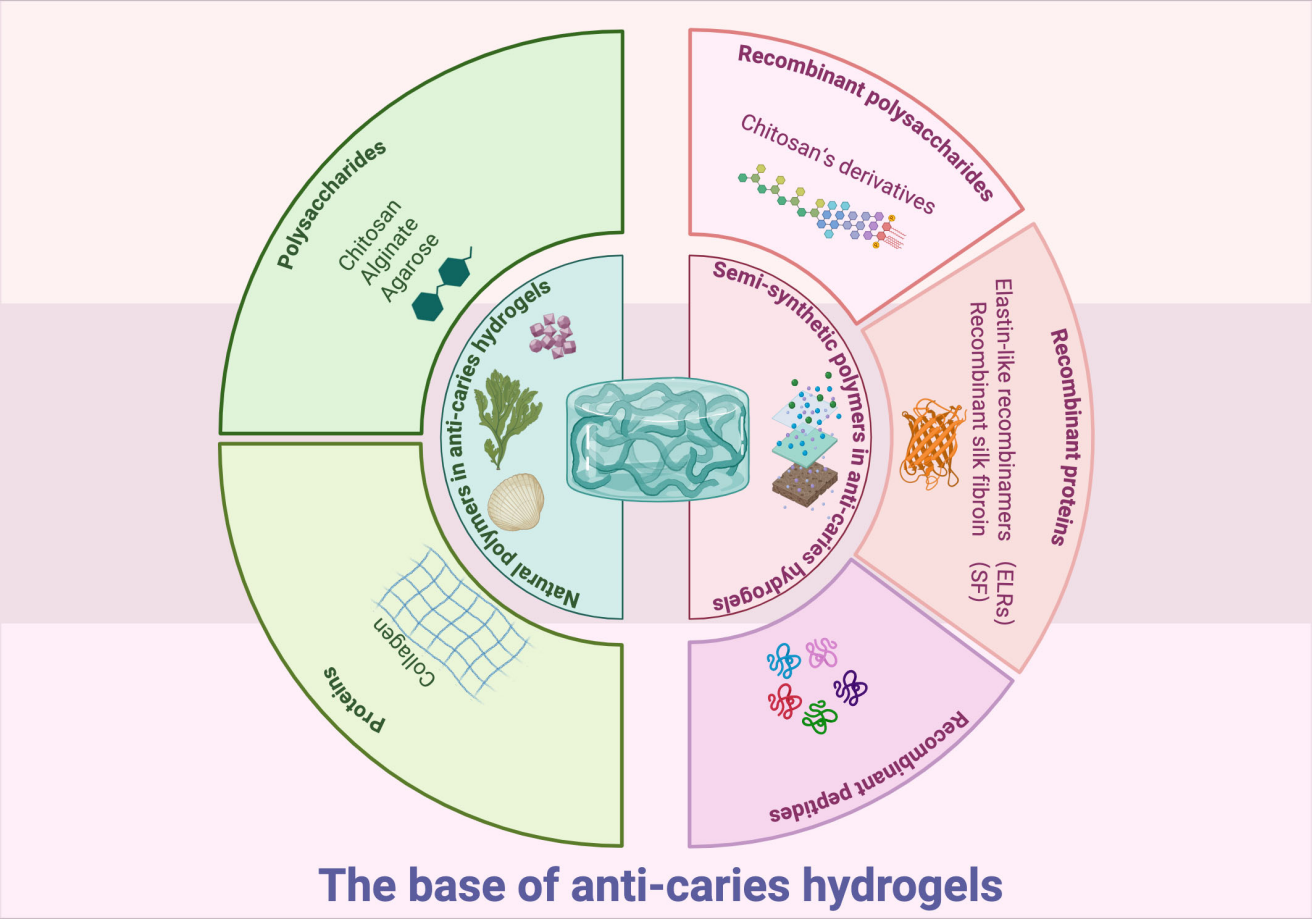
Graphical summery of the base of anti-caries hydrogels. Created in BioRender. Yuqing, C. (2025) https://BioRender.com/1phjnk0.

Commonly, gelling materials serve as the hydrogel base, functioning as the structure matrix. The matrix mainly acts as a carrier for effective ingredients (Cao et al., 2021). Hydrogel bases may comprise natural, synthetic or semi-synthetic polymers (**Figure 1**) (Taghipour et al., 2020). At present, only natural and semi-synthetic hydrogels are utilized in preventing or treating dental caries.

### 2.1 Natural polymers in anti-caries hydrogels

Natural polymers, such as polysaccharides (chitosan, alginate, and agarose) and proteins (collagen), have been studied as structural matrix for anti-caries hydrogels (Estes Bright et al., 2022; Rafiee et al., 2024; Mukherjee et al., 2021). As natural polymer matrix originated from living organisms, they exhibit good biocompatibility and accessibility (Coviello et al., 2007). However, many natural polymers lack inherent antibacterial efficacy (Zhong et al., 2020). The past decades have seen many researches incorporate antibiotic and antibacterial agents into natural polymer-based hydrogels to improve antibacterial properties (Zhong et al., 2020). Furthermore, cross-linking these hydrogels with other polymers has been demonstrated to improve stability, tissue adhesion, and mechanical properties (Zhen et al., 2022; Ju et al., 2023; Yuan et al., 2023; Mohanto et al., 2023; Hu et al., 2019). These advancements position natural polymer hydrogels as promising candidates for future caries management.

#### 2.1.1 Polysaccharides

Polysaccharide hydrogels possess characteristic of thickening, stability, and gum formation (Tamo et al., 2024). To date, many natural polysaccharides, including chitosan, alginate, and agarose have great potential in making anti-caries hydrogels (Ju et al., 2023).

Chitosan is a natural polysaccharide originated from chitin within exoskeletons of crustaceans (Gholap et al., 2024). Commonly, it is easy to form hydrogels by physically cross-linking with each other, making it widely used (Pellá et al., 2018; Hamedi et al., 2022; Kalantari et al., 2019; Gholap et al., 2024). These applicable capabilities might originate from its excellent biocompatibility, biodegradability, antibacterial ability, and cell affinity (Pellá et al., 2018; Hamedi et al., 2022; Kalantari et al., 2019; Gholap et al., 2024). Due to equipped with positive charged amine groups, many chitosan-based hydrogels realize loading or releasing materials they carried, through charge interactions with other negative charged amine groups (Sereni et al., 2017; Pellá et al., 2018; Mohamed et al., 2017). Given all these features, chitosan-based hydrogels can serve as effective carriers of antibacterial or remineralization agents for prevention and treatment of dental caries. In addition, chitosan has the ability to suppress the growth of certain oral bacteria, like *Streptococcus mutans* (*S. mutans)* and *Porphyromonas gingivalis* (*P. gingivalis*), and inhibit the plaque formation (Li et al., 2020; Veltman et al., 2022). Flexibility of chitosan allows it bind to bacteria, penetrate cell membranes, alter DNA structure, prevent the formation of biological macromolecules, and reduce the production of virulence factors in pathogenic bacteria (Li et al., 2022a).

Alginate, mainly obtained from seaweeds, shows promising property in forming ionic hydrogels through coordination between polyvalent metal ions and alginate macromolecules, following the exchange of alginate ions with polyvalent cations (Zhang et al., 2021a). It also shows remarkable advantages like biocompatibility, porosity, adjustable viscosity and water retention capacity (Liu et al., 2022a). All of these features make it an ideal material for biomedical applications (Maity and Das, 2021; Song et al., 2023). At present, alginate-based hydrogels, like sodium alginate synthetic hydrogel, have been applied to remineralize hard tissues of teeth. They show excellent delivering talents, effective mineralization-inducing abilities, and even ion-triggered self-healing abilities (Zhang et al., 2021c; Estes Bright et al., 2022). However, the uncontrolled dissolution of the alginate polymer network, caused by ion exchange or loss, remains a non-negligible problem (Kharkar et al., 2013). More researches still remain to be carried out dealing with these issues.

Agarose, a water-soluble polysaccharide, is notable for its crosslinking ability and effectiveness in delivery properties (Khodadadi Yazdi et al., 2020). It has ability to expand within aqueous solution, allowing agarose-based hydrogels to load drugs when swollen, thereby maximizing drug diffusion (Kolanthai et al., 2017). Under physiological conditions, agarose-based hydrogel can mimic gel-like organic matrix environment in controlling the size and form of hydroxyapatite (HAP) crystals via interacting with calcium by hydroxyl group of agarose (Cao et al., 2014). What’s more, it can also carry remineralization-inducing molecules, making it promising for anti-caries therapy (Dong et al., 2021; Khodadadi Yazdi et al., 2020). Nowadays, studies investigated the role of agarose-based gels in tooth remineralization, demonstrated that agarose hydrogel could replenish mineral precursors and control the size of calcium phosphate complex. Han et al. invented a novel agarose hydrogel system and successfully remineralized dentin *in vivo* (Han et al., 2017). Besides, Moshy et al. induced remineralizaton via agarose-based hydrogel on the caries lesion with better biosafety (El Moshy et al., 2018). With advancements in agarose-based hydrogels, researchers have successfully translated these biomaterials from *in vitro* biomimetic models to *in vivo* rabbit models, demonstrating significant efficacy in dental caries treatment.

#### 2.1.2 Proteins

Proteins are organic macromolecules characterized by distinct three-dimensional conformations formed through the folding and coiling of polypeptide chains. These chains consistently adopt specific spatial configurations essential for biological function. The covalent and non-covalent aggregations of proteins, like collagens, facilitate the formation of gel networks. These protein-based hydrogels equip with certain stability and elasticity (Fu et al., 2023a; Zheng and Zuo, 2021; Antoine et al., 2014). While protein-based hydrogels have shown promise for dental hard tissue remineralization, their unmodified forms are rarely employed in caries management.

Collagen, which can be widely founded in the extracellular matrix, is the most common protein in the human body (Knudsen et al., 1985; Morris-Wiman and Brinkley, 1992; Singh et al., 1998). It is also crucial as the basic component of teeth tissues (Väänänen et al., 2004; Tabata et al., 1995; Lukinmaa and Waltimo, 1992). It can cross-link through chemical, physical or enzymatic way to form hydrogels (Geanaliu-Nicolae and Andronescu, 2020). Upon hydrogel formation, the material exhibits significantly enhanced stability, mechanical strength, and toughness, positioning it as an advanced biomaterial for dental applications (Yang et al., 2022). Moreover, its distinctive triple-helix structure confers molecular recognition capabilities that facilitate efficient carrier functionality (Ruszczak and Friess, 2003; An et al., 2016). At present, collagen-based hydrogel is widely used for remineralization and anti-bacterial activities (Fu et al., 2023b; Amaya-Chantaca et al., 2023; Ikeda et al., 2022). These collagen-based carriers demonstrate significant efficacy *in vitro* when loaded with therapeutic agents. When loaded with mineralization-inducing or antibacterial agents, collagen-based hydrogels demonstrate good performance *in vitro* for both mineralization and antibacterial activities. For example, odontogenic ameloblast-associated protein (ODAM)-impregnated collagen hydrogel, has been proved to be effective in inducing mineralization (Ikeda et al., 2018).

### 2.2 Semi-synthetic polymers in anti-caries hydrogels

Semi-synthetic polymers are derived from chemical modifications of natural polymers. Recently, hydrogels based on these semi-synthetic polymers have shown promise for caries prevention applications.

#### 2.2.1 Recombinant polysaccharides

Chitosan’s derivatives are good examples. Active groups like amino and hydroxyl groups can be modified by etherification, esterification, crosslinking acetylation, and so on. Modified chitosan derivatives readily form hydrogels through physical cross-linking. These materials constitute ideal anti-caries hydrogels, exhibiting excellent biocompatibility, biodegradability, antibacterial efficacy, and cellular affinity (Pellá et al., 2018; Hamedi et al., 2022; Kalantari et al., 2019; Gholap et al., 2024). These modifications can effectively improve hydrogels’ qualities, including antimicrobial ability, mucoadhesive property, and biocompatibility (Mohire and Yadav, 2010; Dilamian et al., 2013; Arora et al., 2023).

#### 2.2.2 Recombinant proteins

In 2015, Li et al. creatively used a type of special biosynthetic elastin-like recombinamers (ELRs) in templating HAP nanocrystal mineralization (Li et al., 2015). These ELRs are made up of repeating pentapeptide sequences derived from tropoelastin (VPG-Xaa-G) (Arias et al., 2006). The chemical linking procedures modified the features of original polymers, enhancing mechanical stability, elasticity, bioactivity, and self-assembly properties (Li et al., 2015). Then these polymers would undergo monomer polymerization to form hybrid hydrogels. Finally, they successfully employed these ELRs to create synthesized thermo-responsive hydrogels, with promising mineralization-inducing properties (Li et al., 2015).

Recombinant silk fibroin (SF) is another type of chemically cross-linked semi-synthetic hydrogel which can deposit HAP perfectly, and with high tensile biomechanical strength, biocompatibility as well as biodegradability (Belda Marín et al., 2020). In 2022, a study showed that by combining tannic acid (TA), SF, and sodium fluoride (NaF), the composite showed remarkable biological stability and biocompatibility (Zhen et al., 2022). Currently, inspired by hagfish, Zhu et al. developed a fluid-hydrogel conversion system containing silk fibroin-TA-black phosphorene-urea (ST-BP-U) to prevent root caries (Zhu et al., 2024). This hydrogel uniformly coats the root surface and penetrates dentinal tubules. Upon aqueous exposure, it undergoes *in situ* reorganization with enhanced crosslinking density, developing stable wet-adhesion properties. Leveraging this platform, potent phototherapeutic effects and enhanced dentin remineralization are achieved.

#### 2.2.3 Recombinant peptides

Peptides spontaneously self-assemble into ordered structures via non-covalent interactions, enabling their application in drug delivery systems (Liu et al., 2011; Caporale et al., 2021). Besides, some peptides might be effective in inhibiting the growth of cariogenic bacteria, making them the potential candidates for forming anti-caries hydrogels. Sun et al. developed a pH-responsive hydrogel coating, basing on recombinant peptide, aiming at reducing dental caries (Sun et al., 2022). This recombinant peptide-based hydrogel demonstrated significant antibacterial efficacy. However, no comparable recombinant peptide-based hydrogels have been developed for anti-caries applications to date.

In a word, the promising features of semi-synthetic hydrogels in dental fields not only maintain the ideal bioactivity of natural hydrogels but also offer multi-tunable properties derived from various chemical parameters (Park and Park, 2016). However, while many semi-synthetic polymers demonstrate excellent properties in antibacterial activity and remineralization, limited semi-synthetic polymer-based hydrogels are developed for treating dental caries (Itskovich et al., 2021; Barreto et al., 2022; Nistor et al., 2013; Barman et al., 2024). Researches focusing on the effective utilization of semi-synthetic polymer-based hybrid hydrogels for caries treatment remains largely unexplored. Further studies are needed to address this gap.

## 3 Hydrogels for prevention and treatment of caries

**Figure 2:**
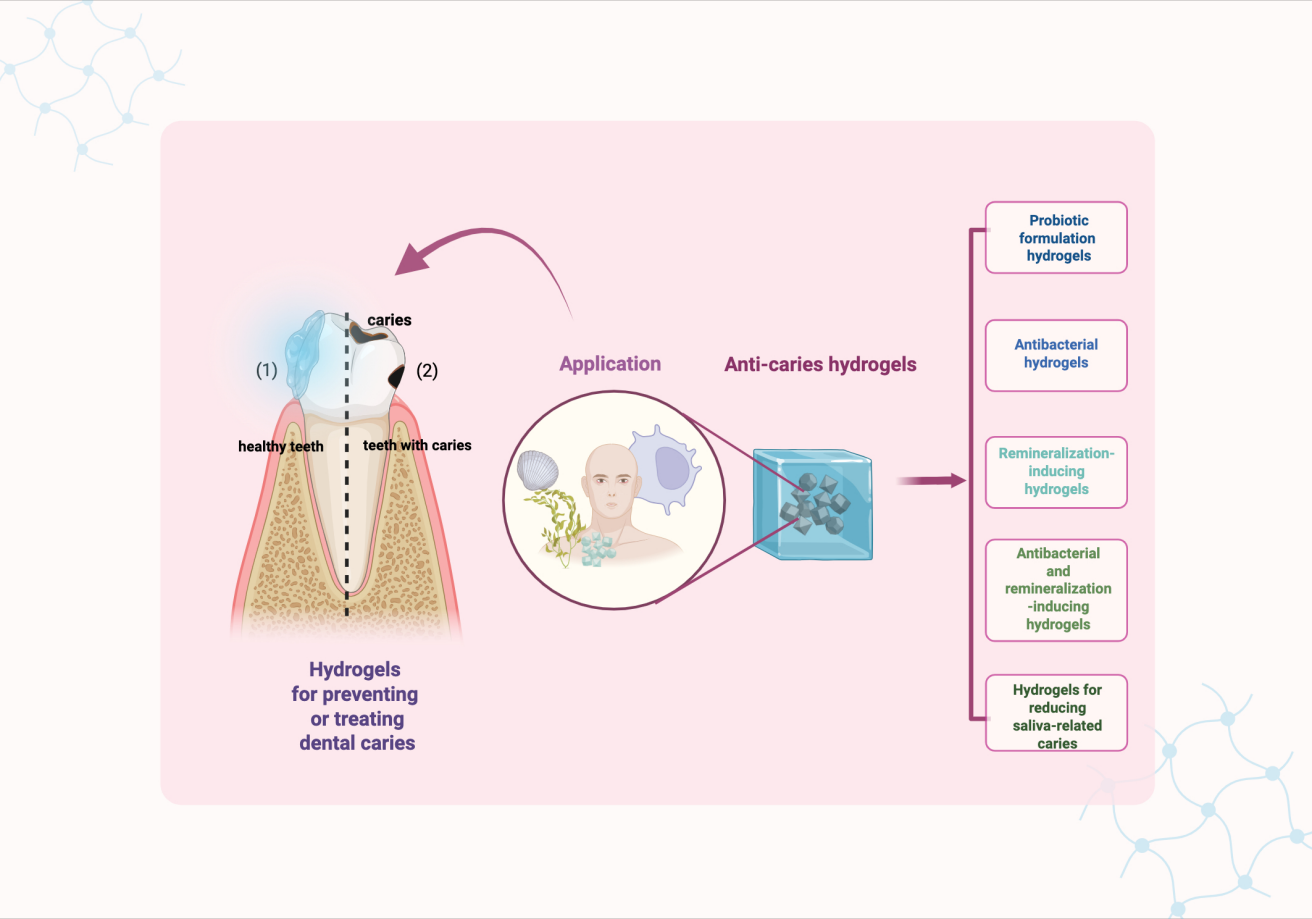
Graphical summery of anti-caries hydrogels. Created in BioRender. Yuqing, C. (2025) https://BioRender.com/uhrig6b.

Generally, hydrogels used for preventing and treating dental caries can be classified in to five types: probiotic formulation hydrogels, antibacterial hydrogel, remineralization-inducing hydrogel, antibacterial and remineralization-inducing hydrogel, and salivary-gland-regenerating hydrogel (**Figure 2**).

### 3.1 Probiotic formulation hydrogels

Dental biofilms are highly assembled microbial communities, surrounded by an extracellular matrix that includes both commensal bacteria and opportunistic pathogens. Under healthy oral conditions, these components maintain a relative balance (Bowen et al., 2018; Gao et al., 2018; Takahashi and Nyvad, 2011). However, once the balance was broken, like cariogenic bacteria abnormal increasing, there will be higher risk to gain dental caries (Takahashi and Nyvad, 2011). Nowadays, a novel treatment known as oral microbiome transplantation (OMT) has been developed, and might have potential to be used for clinical treatment in the future (Weyrich et al., 2024; Beikler et al., 2021; Xiao et al., 2021; Huang and Cheng, 2024). The primary aim of OMT is to address oral dysbiosis, restore ecological balance, and maintain stable homeostasis with the host’s immune system. This can be achieved by using hydrogel delivery vehicle to introduce samples containing the healthiest microbiota into the oral environment. An OMT has been applied among rats and mice successfully (Nath et al., 2021). This method is emerging as a promising therapeutic strategy for dental caries prevention and management, though further validation through clinical studies is warranted.

Recent studies have indicated that *Candida albicans* (*C. albicans*) has the potential to contribute to dental caries. So, an experiment by Ribeiro et al. focused on inhibiting cariogenic *C. albicans* using gellan *gum-Lactobacillus* sp*ecies* (*Lactobacillus* sp.) cells (Ribeiro et al., 2020). This study marked the first investigation that utilized gellan gum hydrogel to incorporate *Lactobacillus* sp. cells to control oral candidiasis, which is relevant to inducing caries. In another study, Ribeiro et al. selected the non-toxic natural polysaccharide gellan gum as a delivery vehicle to deliver *Lacticaseibacillusparacasei* (*L. paracasei*)28.4 into the oral cavity, aiming to inhibit *C. albicans* growth and development of candidiasis. The gellan gum protected drug inside from degradation caused by physical and chemical factors in oral environments. *In vitro* experiments demonstrated that the bacteria released from the probiotic-gellan gum formulation effectively inhibited *C. albicans* growth and biofilm formation on acrylic resin surfaces. Additionally, *in vivo* experiments using a murine model confirmed that the probiotic-loaded gellan gum at concentrations of 1% (wt/vol) and 0.6% (wt/vol) facilitated oral colonization by *L. paracasei*, which was sufficient to prevent *C. albicans* growth and the associated symptomatic lesions like dental caries.

### 3.2 Antibacterial hydrogels

Colonization, growth and metabolism of cariogenic bacteria are important factors leading to dental caries (Selwitz et al., 2007). A lot of hydrogel materials for suppressing the caries pathogenic bacteria have been developed. These hydrogels can kill the cariogenic bacteria by incorporated molecules such as peptides, reactive oxygen species (ROS), certain enzyme and antibody (Luong et al., 2022; Li et al., 2022b; Patel, 2020).

Metal and metallic oxides have been used in controlling dental caries for quite a long time because of their outstanding features in interfering with bacterial metabolism and preventing biofilm formation. Metal ions induce both oxidative stress and non-oxidative mechanisms to realize significant antimicrobial effect (Nizami et al., 2021). Hydrogels combined with ions or oxides of silver, zinc, titanium, and copper have shown effective in preventing dental caries. Afrasiabi et al. demonstrated the potential of zinc oxide nanoparticles (ZnO NPs) doped into a zeolite/chitosan hydrogel (ZnONC-CS) for preventing dental caries (Afrasiabi et al., 2021). ZnONC-CS gel significantly inhibited *S. mutans* growth, affected its metabolic activity, and also effectively decreased the expression of the gtfB, gtfC, and ftf genes, making it a promising candidate for caries management. In 2022, Li et al. reported an intelligent fast-cross-linking hydrogel activated by green light. Their experiments demonstrated that Bi_12_O_17_Cl_2_ and Cu_2_O rapidly cross-link with calcium ions (Ca^2+^) to form an adhesive network structure on tooth surfaces. This structure releases ROS upon green light exposure, inducing localized sterilization, biofilm removal, and caries prevention in both *in vitro* and *in vivo* models (Li et al., 2022b).

Glucosyltransferases (Gtfs) are enzymes involved in the pathogenic proliferations and actions of *S. mutans* to mediate dental caries (Zhang et al., 2021b). They induce biofilm formation, leading to the colonization of cariogenic bacteria. Therefore, Gtfs are considered an important target for inhibiting cariogenic biofilms. Ahirwar et al. modified the structure of a biofilm inhibitor IIIC5 to synthesize a novel pH-responsive hydrogel: hydroxy aurones 5 (HA5) (Ahirwar et al., 2023). This compound inhibited Gtfs and suppressed glucan production. Moreover, HA5 could selectively inhibit *S. mutans* biofilms and reduce caries incidence, demonstrating its potential as an ideal candidate to address traditional challenges of anti-caries drugs. These challenges include limited solubility, poor biofilms penetration, and inability to retain efficacy in infected areas within the complex oral cavity environment.

### 3.3 Remineralization-inducing hydrogels

#### 3.3.1 Demineralization and remineralization balance in caries

In oral environment, the balance between demineralization and remineralization of teeth hard tissues is significant to caries (Selwitz et al., 2007). The emergence, progression, cessation, or reemergence of caries largely depends on whether this balance is preserved (Featherstone, 2004). When the balance was broken and demineralization becomes the predominant, the process of dental caries formation is stimulated (Selwitz et al., 2007; Wang et al., 2021). Consequently, the remineralization of demineralized teeth has long been a major research focus in dental science. Some remineralization-inducing hydrogels function by directly or indirectly interacting with Ca²^+^, PO_4_³^-^, and HAP to promote stable mineral deposition on the tooth surface (**Table 1**). The resulting mineralized layers cover both intact surfaces and demineralized regions, thereby protecting dental hard tissues from acid erosion (Parker et al., 2014; Sun et al., 2014).

**Table 1:** Overview of remineralization-inducing hydrogels.

Name	Type	Active ingredients	Result	Year	Ref.
NaF/chitosan hydrogel	Fluoride hydrogel	NaF	This hydrogel restored enamel-like prismatic tissue and controlled the formation, size, and mineral composition of crystals through synergistic interactions with calcium, phosphate, and fluoride ions. The regenerated apatite crystals showed strong c axis orientation and high crystallinity.	2014	(Cao et al., 2014)
Mussel-inspired wet adhesive fluoride system (denoted TS@NaF)		NaF	TS@NaF exhibited biological stability and biocompatibility. It showed consistent wet adhesion, and released fluoride ions (F-), resulting in significant calcium fluoride (CaF_2_) deposition on enamel *in vitro*. Additionally, TS@NaF demonstrated an anti-caries effect and increased enamel mineral density.	2022	(Zhen et al., 2022)
NaF hydrogel coating		NaF	This fluoride gel effectively reduced early root surface caries in demineralized dentin by enhancing fluoride uptake and delivering fluorine to deeper dentin before pH cycling. Moreover, it was capable of supplying fluorine into the dentin and preventing dentin demineralization.	2022	(Matsuda et al., 2022)
NaF hydroxyapatite nanocomposite hydrogel		NaF	The 70/30 (HAp-CS) nanocomposite gel effectively increased the microhardness and mineral composition of lesions demineralized for 24 hours. But more severe lesions require stronger formulations or longer application times.	2024	(Fathy et al., 2024)
EMD-calcium chloride (CaCl_2_) agarose hydrogel	Bioactive factor-loaded hydrogel	Enamel matrix derivative (EMD), calcium chloride (CaCl_2_), and fluoride	The hydrogel system successfully facilitated *in vivo* regeneration of HAP crystals, with structural and mechanical properties comparable to natural dentin, on damaged dental tissues.	2017	(Han et al., 2017)
Odontogenic ameloblast-associated protein (ODAM)-impregnated collagen hydrogel		ODAM	The hydrogel resulted in formation of calcium phosphate precipitates, after treated 24 hours. Transmission electron microscopy and selected area electron diffraction analyses showed these crystals consisted of needle-like HAP.	2018	(Ikeda et al., 2018)

#### 3.3.2 Fluoride hydrogels

Historically, fluoride was the first practical approach for caries prevention due to its ability to induce remineralization (Zampetti and Scribante, 2020). However, traditional fluoride applications faced limitations imposed by the wet, dynamic oral environment, which compromised delivery efficiency and rapidly cleared drugs from tooth surfaces. These challenges were subsequently addressed through the development of fluoride hydrogel (Zhen et al., 2022). Fluoride ions released from the hydrogels interact with dental HAP to form acid-resistant fluorapatite (Bossù et al., 2019).

In 2014, Cao et al. firstly developed an effective NaF/chitosan hydrogel (Cao et al., 2014). This hydrogel restored prismatic enamel-like tissue while controlling formation, size, and mineral composition. It induced remineralization through synergistic interactions with calcium, phosphate, and fluoride ions, producing regenerated apatite crystals with strong c-axis orientation and high crystallinity. Contemporary fluoride hydrogel development has evolved from simple NaF encapsulation to advanced formulations designed for moist oral environments and physiological temperature fluctuations. These hydrogels sustain fluoride release, providing long-term anticaries and antibacterial effects with improved biocompatibility and stability. Matsuda et al. demonstrated that fluoride hydrogels significantly increase fluoride uptake on acid-etched root surfaces and inhibit hard tissue demineralization. For improved performance in moist oral conditions (Matsuda et al., 2022). For better application in moist and dynamic oral cavity environment, another study pioneered a mussel-inspired wet adhesive self-assembled fluoride hydrogel (TS@NaF) (Zhen et al., 2022). Zhen et al. used *in vitro* retention and degradation tests to simulate the drug performance under complex oral conditions. They proved this material could ideally resist the attack of enzymes and washing of liquids. This system exhibits superior stability, biocompatibility, and promotes effective calcium fluoride deposition on enamel.

However, while fluoride effectively combats caries, excessive fluoride intake poses risks like dental fluorosis, especially in children. As the primary source of fluoride exposure, community water fluoridation targets a concentration of 1 ppm. This specific level is chosen to optimize fluoride’s cavity-preventing benefits while minimizing the risk of dental fluorosis (Buzalaf and Levy, 2011). Moreover, prolonged fluoride exposure may lead to the development of fluoride-resistant bacterial strains. Such strains could disrupt the microecological balance of biofilms and reduce their *in vitro* anti-caries efficacy (Shen et al., 2022). Therefore, precise control of fluoride concentration is essential in future application of fluoride hydrogels.

#### 3.3.3 Bioactive factor-loaded hydrogels

Contemporary developments in remineralization hydrogels carrying bioactive factors represent a promising strategy for caries prevention. The mechanisms of caries formation underscore the relationship between bioactive factors and mineralization processes (Selwitz et al., 2007). Utilizing hydrogels to deliver remineralization-promoting bioactive factors offers a novel therapeutic approach. And lots of studies focus on enamel matrix derivative (EMD) and ODAM-impregnated collagen systems.

The effects of EMD on biomimetic mineralization were first reported a decay ago. Cao et al. pioneered its application for enamel remineralization using an EMD-calcium chloride (CaCl_2_) agarose hydrogel. A 2 mm-thick layer of the EMD-CaCl_2_ hydrogel was applied onto demineralized enamel slices, overlaying by ion-free agarose and phosphate solution with fluoride. This system mediated *in vitro* mineralization of human enamel, with subsequent optimizations advanced EMD hydrogels efficacy (Han et al., 2017).

Amelotin (AMTN) is a secreted proteins in human body which can induce enamel biomineralization (Somogyi-Ganss et al., 2012; Nishio et al., 2011). Over the past few decades, researches have demonstrated the enamel mineralization inducing ability of AMTN-related complexes (Gasse et al., 2012; Núñez et al., 2016). ODAM, expressed during ameloblast maturation, strongly interacts with AMTN under physiological conditions (Nishio et al., 2013). Ikeda et al. investigated ODAM nucleation in collagen matrics after 24-hour incubation in ODAM-impregnated collagen hydrogel in simulated body fluid (SBF) buffer. They found the composite hydrogel promotes HAP nucleation both in SBF and in non-biological environments, with dose-dependent ways (Ikeda et al., 2018).

### 3.4 Antibacterial and remineralization-inducing hydrogels

The optimal strategy for caries prevention and treatment involves dual-action anti-caries hydrogels that simultaneously inhibit cariogenic bacteria and promote remineralization (**Table 2**). Currently, amelogenin peptide-based hydrogels represent the most extensively studied and clinically promising formulation.

**Table 2:** Antibacterial and remineralization-inducing hydrogels.

Name	Type	Active ingredients	Result	Year	Ref.
Chitosan-QP5 hydrogel	Amelogenin peptide hydrogel	QP5	Chitosan-QP5 hydrogel inhibited *S. mutans* (MIC 5 mg/mL, 95% antibiofilm), and the enhanced enamel remineralization (50% hardness recovery) and pH-responsive *in vitro* efficacy were confirmed.	2019	(Ren et al., 2019; Liu et al., 2022b)
Amelogenesis-inspired QP5 bioactive glass (BG) hydrogel		QP5, BG	The QP5 released from hydrogel triggered enamel remineralization by guiding the release of Ca^2+^ and PO4^3-^ from BG. Showed excellent antibacterial effects and remineralization abilities of initial carious lesions.	2022	(Liu et al., 2022b)
rP172-releasing hydrogel		rP172, Ca^2+^, phosphate, and fluoride	This amelogenin-hydrogel significantly increased enamel microhardness in artificial caries (static/pH/biofilm models), and reduced mineral loss with no cytotoxicity to periodontal cells.	2012	(Fan et al., 2012)
P26-chitosan (P26-CS) hydrogel and P32-chitosan (P32-CS) hydrogel		P26, P32, and chitosan	A denser coating of organized hydroxyapatite (HAP) crystals was formed covering entire demineralized enamel surfaces after treated with P26-CS and P32-CS hydrogels. And hardness and modulus of enamel were increased with no significant difference in the mechanical properties between the two peptide hydrogels.	2021	(Mukherjee et al., 2021)
P26-CS hydrogel and P32-CS hydrogel		P26, P32, and chitosan	The hydrogels ideally improved microstructure, mineral density, mineral phase, and nanomechanical properties of hard tissues.	2023	(Cai and Moradian-Oldak, 2023)
Dual-function Pluronic F127-alginate hydroge	Fluoride hydrogel	NaF and S-nitrosoglutathione (a nitric oxide donor)	The hydrogel reduced 98% micro-bacteria and effectively inhibited the demineralization of enamel-like substrates under acidic conditions.	2022	(Estes Bright et al., 2022; Ikeda et al., 2018)

Hydrogels carry amelogenin peptides were proved effective in antibacteria and remineralization inducing. The hydrogel containing QP5 is a good example. QP5 is effective in repairing the demineralized teeth by inducing remineralization (Ding et al., 2020). Ren et al. combined amelogenin-derived peptide QP5 with chitosan to develop a novel chitosan-QP5 hydrogel, demonstrating its long-term inhibitory effects on *S. mutans* biofilm growth, lactic acid production and metabolic activity (Ren et al., 2019; Liu et al., 2022b). Later, Liu et al. synthesized an amelogenesis-inspired hydrogel composite incorporating QP5 peptide and bioactive glass (BG) (Liu et al., 2022b). Their results revealed that QP5 promote enamel remineralization by directing the release of Ca^2+^ and PO4^3-^ from BG. Therefore, chitosan-QP5 hydrogels represent promising candidates for caries control, owing to their antibacterial and remineralization abilities.

Beyond QP5, additional amelogenin peptide-chitosan hydrogels incorporating rP172, P26 and P32 have been studied. Fan et al. demonstrated rP172-releasing hydrogel loaded with Ca^2+^, phosphate, and fluoride improved enamel microhardness while exhibiting no cytotoxicity to periodontal ligament cells (Fan et al., 2012). Mukherjee et al. induced enamel remineralization with increased hardness by using P26-chitosan (P26-CS) and P32-chitosan (P32-CS) hydrogels (Mukherjee et al., 2021). In 2023, Cai et al. further verified the *in situ* remineralization inducing ability of P26-CS and P32-CS hydrogels (Cai and Moradian-Oldak, 2023). The results revealed that these hydrogels effectively improved the microstructure, mineral density, crystalline phase composition, and nanomechanical properties of dental hard tissues.

Fluoride-containing hydrogels demonstrate dual efficacy when combined with complementary antibacterial and remineralizing components. Bright et al. engineered a dual-functional Pluronic F127-alginate hydrogel incorporating NaF and S-nitrosoglutathione (Estes Bright et al., 2022). The hydrogel elicited nearly 98% viable bacteria while effectively inhibited the demineralization of enamel-like substrates under acidic conditions.

### 3.5 Hydrogels for reducing saliva-related caries

**Figure 3:**
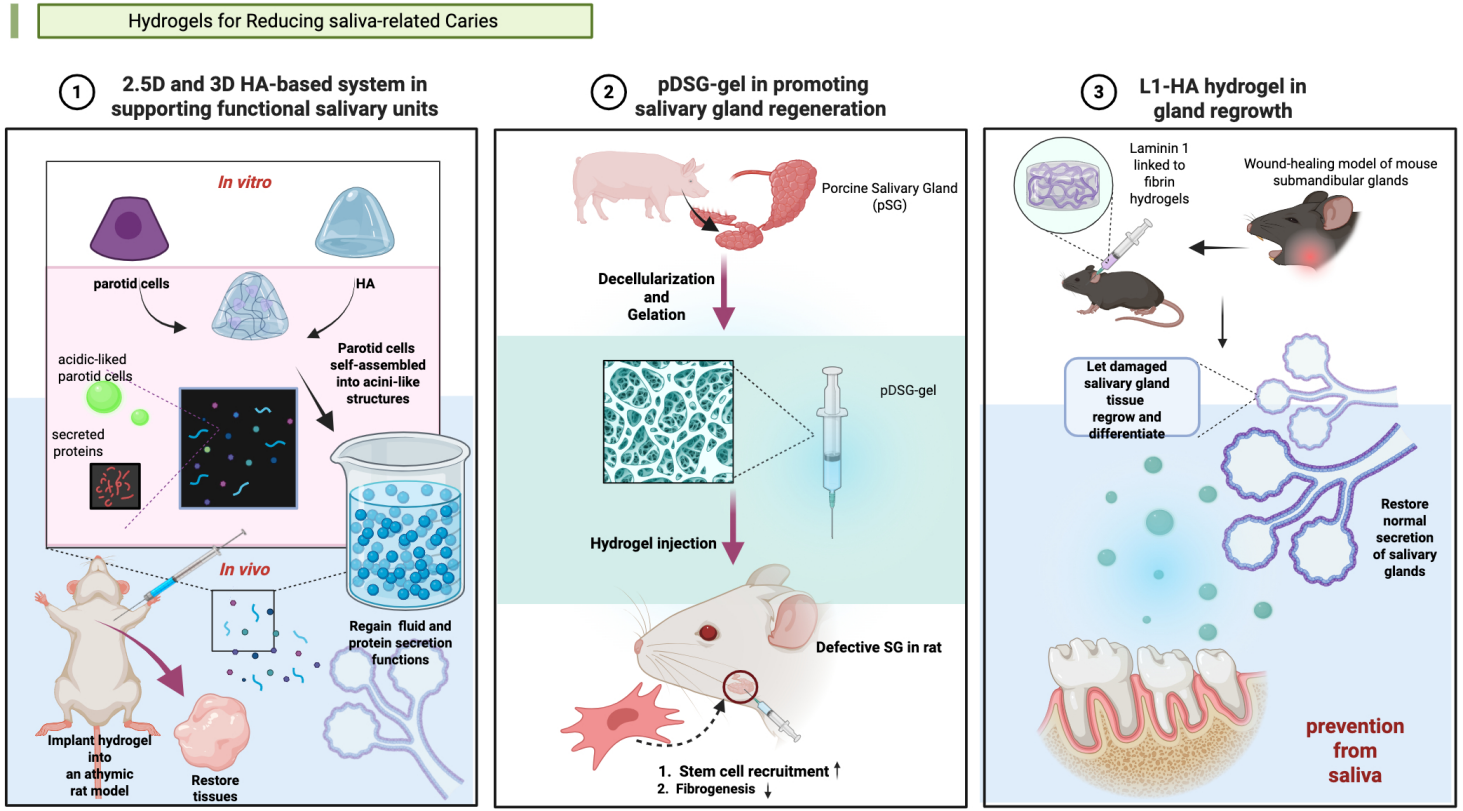
Hydrogels applied in salivary gland (SG) tissue engineering. (1) Synthesis of a 2.5-dimensional (2.5D) and a three-dimensional (3D) hyaluronic acid (HA)-based culture system in supporting functional salivary units (Pradhan-Bhatt et al., 2013). (2) Synthesis of a pDSG-gel in promoting salivary gland regeneration (Wang et al., 2023a). (3) Synthesis of a L1-HA hydrogel in gland regrowth (Nam et al., 2017). Created in BioRender. Yuqing, C. (2025) https://BioRender.com/qow0ggb.

The constituents and properties of saliva play an essential role in the occurrence and progression of dental caries. As one of the most important host factors, Saliva mediates the cariogenic process (Lenander-Lumikari and Loimaranta, 2000). However, under specific pathological conditions, including post-radiotherapy/chemotherapy, autoimmune diseases which salivary gland dysfunction can occur, altering saliva composition and reducing secretion. These changes may collectively contribute to caries formation (Tappuni and Challacombe, 1994; Mansson-Rahemtulla et al., 1987; Lenander-Lumikari and Loimaranta, 2000). Hydrogel materials are now developed basing on this theory to prevent caries (**Figure 3**).

Some hydrogels manage caries via importing salivary substances. In 2013, Pradhan-Bhatt et al. developed a three-dimensional (3D) hyaluronic acid (HA)-based hydrogel culture system that supports salivary functional units (Pradhan-Bhatt et al., 2013). And the good structural integrity and long viability of the 3D hydrogel *in vitro* and *in vivo* was confirmed (Pradhan-Bhatt et al., 2013). The hydrogel worked like natural salivary, conducting anti-caries effectiveness.

Hydrogels can promote salivary gland regeneration showed potential of caries risk reduction. In 2017, Nam et al. pioneered this approach by developing L1 Peptide-conjugated fibrin hydrogels to promote the regeneration of salivary glands (Nam et al., 2017). Wang et al. engineered an injectable decellularized extracellular matrix hydrogel to prevent dental caries through salivary glands regeneration (Wang et al., 2023). Currently, few studies have explored this therapeutic strategy for caries prevention and treatment. The precise relationship between regeneration-induced saliva secretion and caries prevention efficacy remains to be fully elucidated.

## 4 Conclusion

Anti-caries hydrogels represent a critical frontier in preventive dentistry, with contemporary efforts focused on functionalized systems encompassing remineralizing, antibacterial, and dual-functional formulations. Currently, chitosan and fluoride are leading hydrogel materials for caries management, owing to their dual capacity to induce remineralization and suppress cariogenic bacteria. However, the complicated oral surroundings remain the major barrier to clinical translation of anti-caries hydrogels. Challenges in maintaining efficacy amid bacterial contamination, saliva dilution, and other interferences are still noticing. To overcome these limitations, many noticeable developments have been made. Emerging innovations, like pH-responsive and thermosensitive intelligent hydrogels enable precision strategies that dynamically respond to the oral microenvironment. Novel approaches including probiotic-loaded hydrogels and saliva-modulating systems demonstrate transformative potential, though further mechanistic validation remains essential for clinical translation.

It is worth noting that a critical limitation of multifunctional hydrogels lies in their potential biocompatibility concerns. Therefore, further research should prioritize developing non-cytotoxic hydrogels systems. Moreover, many current studies lack comprehensive biosafety evaluations and clinical validation. Future studies demand *in vitro* and *in vivo* biocompatibility assessments, and even proceed to clinical trials. In summary, development of hydrogels for caries management remains at an early stage, substantial further investigation is still required.
